# Exercise training attenuates neutrophil infiltration and elastase expression in adipose tissue of high-fat-diet-induced obese mice

**DOI:** 10.14814/phy2.12534

**Published:** 2015-09-04

**Authors:** Noriaki Kawanishi, Hiroyuki Niihara, Tsubasa Mizokami, Koichi Yada, Katsuhiko Suzuki

**Affiliations:** 1 Institute for Nanoscience & Nanotechnology, Waseda UniversityTokyo, Japan; 2 Research Fellow of the Japan Society for the Promotion of SciencesTokyo, Japan; 3 Graduate School of Sport Sciences, Waseda UniversityTokorozawa, Saitama, Japan; 4 Faculty of Sport Sciences, Waseda UniversityTokorozawa, Saitama, Japan

**Keywords:** Elastase, exercise, macrophage, neutrophil, obesity

## Abstract

The innate immune system is associated with the development of local inflammation. Neutrophils play an essential role in the development of the adipose tissue (AT) inflammation associated with obesity by producing elastase, which can promote the activation and infiltration of macrophages. Exercise training attenuates AT inflammation via suppression of macrophage infiltration. However, the mechanisms driving this phenomenon remains to be elucidated. Here, we evaluated the effects of exercise training on the infiltration of neutrophils and elastase expression in an obese mouse model. Four-week-old male C57BL/6J mice were randomly assigned to one of three groups that either received a normal diet (ND) plus sedentary activity (*n *=* *15), a high-fat diet (HFD) plus sedentary activity (*n *=* *15), or a HFD plus exercise training (*n *=* *15). Mice were fed the ND or HFD from the age of 4 weeks until 20 weeks. Mice in the exercise group ran on a treadmill for 60 min/day, 5 days/week over the same experimental period. Mice fed with the HFD had increased content of macrophages in the AT and increased inflammatory cytokine mRNA levels, which were reduced by exercise training. Similarly, AT from the HFD sedentary mice contained more neutrophils than AT from the ND mice, and the amount of neutrophils in this tissue in HFD-fed mice was lowered by exercise training. The mRNA levels of neutrophil elastase in AT were lower in the HFD exercise-trained mice than those in the HFD sedentary mice. These results suggest that exercise training plays a critical role in reducing macrophage infiltration and AT inflammation by regulating the infiltration of neutrophils.

## Introduction

Obesity is associated with a chronic inflammatory state, which in turn has been largely attributed to local tissue inflammation in the visceral adipose tissue (AT) and liver (Gustafson et al. 2007). An excessive inflammatory response in local tissue is considered to be a crucial mechanism for insulin resistance, dyslipidemia, and hypertension associated with obesity (Cornier et al. 2008). Recently, the innate immune system has been identified to be associated with the development of local inflammation (Chmelar et al. 2013; Mathis 2013). Macrophages residing in AT, liver, and skeletal muscle release inflammatory cytokines and reactive oxygen species, which in turn induce organ dysfunction (Lumeng and Saltiel 2011). A recent study demonstrated that obesity not only induces infiltration of macrophages into AT, but also changes in the subset of macrophages present (Lumeng et al. 2007). Two subsets of macrophages have been operationally defined: M1 macrophages, which exhibit a proinflammatory activation profile and M2 macrophages, which possess a more immunosuppressive profile. The presence of M1 macrophages is increased in AT during obesity, and they are a primary driver of inflammation (Mantovani et al. 2004).

Recently, neutrophils have also been shown to play an essential role in the development of AT inflammation. In human obesity, increased infiltration of neutrophils into AT was observed (Rouault et al. 2013). Recent evidence has also indicated that administration of a high-fat diet (HFD) increased the cell number of neutrophils in murine AT (Carmon et al. 2008). Neutrophils produce several proteases, which can promote the activation and infiltration of macrophages. Interestingly, Talukdar et al. (2012) reported that neutrophil elastase (NE)-deficient obese mice have reduced number of inflammatory macrophages and expression of inflammatory cytokines in their AT, suggesting that NE may be of central importance in the development of AT inflammation.

Exercise has anti-inflammatory effects and may prevent the development of chronic inflammatory diseases (Gleeson et al. 2011; Lancaster and Febbraio 2014). We and others have shown that high levels of expression of inflammatory cytokines in epididymal visceral AT of mice induced by HFD administration were prevented by endurance exercise training (Vieira et al. 2009a,b; Kawanishi et al. 2010; Linden et al. 2014). We also analyzed the characteristics of the macrophages in epididymal visceral AT by flow cytometry and found that exercise training not only decreased the cell number of macrophages, but also changed the ratio of the macrophage subsets in the AT of obese mice (Kawanishi et al. 2013). These results indicate that chronic endurance exercise may improve AT inflammation by changing the number and subset composition of macrophages. However, the mechanism underlying this phenomenon is yet to be elucidated. Here, we hypothesized that exercise training would decrease AT inflammation and macrophage infiltration via suppression of neutrophil infiltration.

Although AT is classified into subcutaneous fat and visceral fat, visceral AT is more closely related to the development of chronic inflammatory diseases than subcutaneous fat. The largest adipose depot in visceral AT of mice is the epididymal AT; this deposit is specifically increased in mice fed with HFD. Furthermore, epididymal AT has been demonstrated to induce tissue remodeling including neutrophil infiltration along with obesity. Therefore, we have analyzed with a focus on epididymal AT. The purpose of this study was to investigate the effects of exercise training on neutrophil infiltration and elastase expression in AT using HFD-induced obese mice.

## Methods

### Animals, diets, and exercise training protocol

Four-week-old male C57BL/6J mice were purchased from Kiwa Laboratory Animals (Wakayama, Japan). Mice were housed in a controlled environment with a 12 h light–dark cycle. All experimental procedures followed the Guiding Principles for the Care and Use of Animals of the Waseda University Institutional Animal Care and Use Committee (approval number: 2013-A019). The mice were randomly assigned to one of three groups that received either a normal diet (ND) plus sedentary activity (*n *=* *15), a HFD plus sedentary activity (*n *=* *15), or a HFD plus exercise training (*n *=* *15). Although all mice were fed the normal diet up to 4 weeks of age, mice of the HFD plus sedentary group and the HFD plus exercise training group were switched to HFD after 4 weeks of age. The HFD comprised 60% of calories from fat, 20% from protein, and 20% from carbohydrates (D12492; Research Diets, New Brunswick, NJ). The mice were fed the HFD from 4 to 20 weeks of age. The ND mice were fed a standard chow diet consisting of 10% of calories from fat, 20% from protein, and 70% from carbohydrates (D12450B; Research Diets). Mice were provided access to diet and water ad libitum.

Exercise training was initiated at the onset of a HFD, when the mice were 4 weeks old and continued for 16 weeks. Before the experiment, the mice were trained by treadmill running at 15 m/min for 15 min once during the acclimation period. The mice undergoing exercise training were placed on a motorized treadmill (Natsume, Kyoto, Japan) for 60 min/day (during the light phase), 5 days/week. The treadmill speed was set at 15 m/min for the first 4 weeks and 20 m/min for the remaining 12 weeks. Running speed for the first 4 weeks of exercise training was low (15 m/min). In case of this low running speed, mice can perform exercise at the first time of training. Therefore, despite a single day, it is important to confirm that mice were able to complete all of the 60 min session of the running training protocol. While endurance training increases maximal O_2_ uptake, it was not measured in this study. The exercise-trained and untrained mice (20 weeks old) were sacrificed 3 days after the final exercise training session under light anesthesia with inhaled isoflurane (Abbott, Tokyo, Japan). The AT was promptly removed, weighed, frozen in liquid nitrogen, and stored at −80 °C until analysis. Two different types of AT depots were used in the present study. Fat mass of abdominal subcutaneous AT and epididymal AT were measured. Epididymal AT was used for PCR analysis and flow cytometry analysis.

### Isolation of stromal vascular fraction (SVF) cells from AT

Stromal vascular fraction cells were isolated from the epididymal AT (eAT) as described previously (Cho et al. 2014), with some modifications. Briefly, the AT was weighed, minced with scissors, and added to 10 mL of phosphate-buffered saline containing 1 *μ*g/mL heparin (Sigma, St. Louis, MO). The suspension was centrifuged at 800***g*** for 5 min and the pieces of AT floating in the supernatant were collected and placed in 10 mL of pH 7.4 Tyrode's buffer (137 mmol/L NaCl, 5.4 mmol/L KCl, 1.8 mmol/L CaCl_2_, 0.5 mmol/L MgCl_2_, 0.33 mmol/L NaH_2_PO_4_, 5 mmol/L HEPES, and 5 mmol/L glucose) containing 2 mg/mL collagenase type 2 (Worthington, Lakewood, NJ). The mixture was shaken for 20 min at 37 °C, before adding Dulbecco's modified Eagle's medium (Sigma) supplemented with 10% fetal bovine serum to the digested tissue. The sample was filtered through a 70-*μ*m mesh strainer and centrifuged at 800***g*** for 5 min. The pellet containing the SVF cells was resuspended in 2 mL Red Blood Cell Lysis buffer (Sigma) and filtered through a 40-*μ*m nylon mesh strainer. The isolated SVF cells were washed twice with Staining buffer (BD Pharmingen, Franklin Lakes, NJ) and then counted.

### Isolation of splenocytes

The spleen was removed from the mice and was pressed through 70-*μ*m cell strainers using a syringe barrel. The resulting cell suspension was centrifuged at 800***g*** for 5 min, the cell pellet was resuspended in 2 mL of Red Blood Cell Lysis buffer, and the suspension was filtered through a 40-*μ*m nylon mesh strainer. The cells were washed twice with Staining buffer and counted using a Scepter Handheld Automated Cell Counter (Millipore, Long Beach, CA).

### Flow cytometry analysis

The SVF cells and splenocytes (2.5 × 10^5^ cells/sample) were incubated with Fc-blocker (anti-CD16/CD32; eBioscience, San Diego, CA) for 20 min and then stained with combinations of anti-CD11b PE-Cy7, anti-Ly-6G PE-Cy5, and anti-F4/80 PE-Cy5, anti-CD11c PE for 20 min. Heparin-treated whole blood cells (50 *μ*L) were stained with anti-CD11b PE-Cy7, anti-Ly-6G PE-Cy5, anti-F4/80 PE-Cy5, or anti-CD11c PE (all from eBioscience). After 20-min incubation in the dark, whole blood cells were incubated with 600 *μ*L Versa Lyse (Beckman Coulter, Tokyo, Japan) at room temperature in the dark. The SVF cells, splenocytes, and blood cells were washed with Staining buffer (BD Pharmingen). Finally, cells were resuspended in Staining buffer and analyzed using fluorescence-activated cell sorting analysis performed with Guava® EasyCyte™ 6HT flow cytometry system (Millipore, Long Beach, CA) and InCyte software (Millipore). A validation of the flow cytometric approach for the identification of total CD11b^+^ Ly-6G^+^ neutrophils is shown in Supplementary Figure S1.

### Real-time quantitative polymerase chain reaction

We analyzed the mRNA levels in eAT using our previously described methods (Kawanishi et al. 2010). Gene expression was normalized to the housekeeping gene glyceraldehyde-3-phosphate dehydrogenase (GAPDH). All data in mRNA levels are represented relative to its expression (i.e., using standard curve methods) as fold changes from the ND plus sedentary group. The sequences of the primer pairs are shown in Supplementary Table S1.

### Statistical analyses

All data are expressed as mean ± SEM. Statistical analyses were performed using the Statistical Package for the Social Sciences (Version 18.0; SPSS Inc., Chicago, IL). Multiple comparisons were performed using Tukey's post hoc tests after one-way analysis of variance. The level of significance was set at *P *<* *0.05.

## Results

### Effects of HFD and exercise training on body weight and body composition

Table 1 shows the body mass, fat mass, and liver mass of each group of mice. The body mass, subcutaneous fat mass, and liver mass in the HFD sedentary mice were greater than the ND sedentary and the HFD exercise mice (Table 1). The epididymal fat mass and liver mass was greater in the HFD sedentary mice than in the ND sedentary mice (Table 1), but this difference was not observed between the HFD sedentary mice and the HFD exercise mice. Caloric intake, which was calculated from the diet and food consumption, was higher in the HFD sedentary mice than in the ND sedentary mice, but caloric intake was not significantly affected by exercise.

**Table 1:** Comparison of body mass, fat mass, liver mass, and caloric intake between normal diet (ND) and high-fat diet (HFD) in sedentary and exercise mice

	ND	HFD	
	Sedentary	Sedentary	Exercise
Initial body mass (g)	16.3 ± 0.2	16.3 ± 0.1	16.6 ± 0.2
Final body mass (g)	34.2 ± 0.7	49.9 ± 1.2^a^	41.9 ± 1.3^a^,^b^
Epididymal fat mass (g)	1.65 ± 0.09	2.41 ± 0.16^a^	2.21 ± 0.21^a^
Abdominal subcutaneous fat mass (g)	1.28 ± 0.09	3.51 ± 0.15^a^	2.11 ± 0.25^a^^,^^b^
Liver mass (g)	2.08 ± 0.07	3.17 ± 0.18^a^	2.24 ± 0.16^b^
Caloric intake (kcal/weeks)	67.3 ± 0.3	72.4 ± 1.5^a^	72.1 ± 1.0^a^

Data are presented as mean ± SEM.

a *P *<* *0.05, different from ND sedentary.

b *P *<* *0.05, different from HFD exercise.

### Effects of HFD and exercise training on macrophage infiltration and AT inflammation

**Figure 1:**
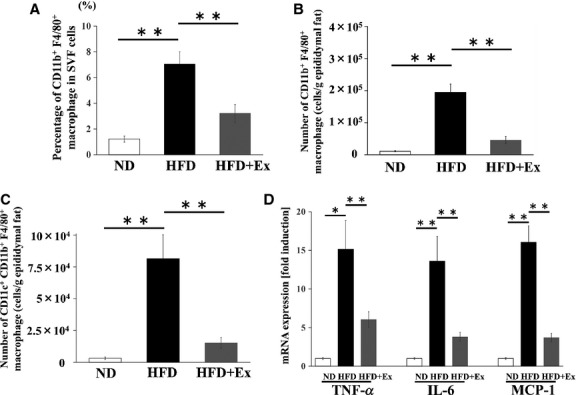
Effect of exercise training on infiltration of macrophages into the visceral adipose tissue and inflammation of normal diet- and high-fat-diet-fed mice. (A) Percentage of CD11b^+^ F4/80^+^ macrophages in stromal vascular fraction (SVF) cells. (B) Number of CD11b^+^ F4/80^+^ macrophages per gram of epididymal adipose tissue (eAT). (C) Number of CD11c^+^ CD11b^+^ F4/80^+^ macrophages per gram of eAT. (D) mRNA expression levels of TNF-*α*, IL-6, and MCP-1 in eAT. Values represent mean ± SEM. *P <* *0.05, **P <* *0.01.

We analyzed the macrophage (CD11b^+^ and F4/80^+^ cells) cell number in the eAT and the percentage of macrophages in SVF cells by flow cytometry. The percentage of macrophages in total SVF cells and the absolute number of macrophages per gram of eAT was higher in the HFD sedentary mice than in the ND sedentary mice (*P *<* *0.01), but was lower in the HFD exercise mice than in the HFD sedentary mice (*P *<* *0.01, Fig. 1A and B). M1 macrophages were characterized by the presence of CD11c, and the cell number of CD11c^+^ macrophages in the eATs in the HFD sedentary mice was higher than the ND sedentary and the HFD exercise mice (*P *<* *0.01, Fig. 1C). Next, we examined the expression of M1 macrophage chemoattractant in the eAT. In the sedentary condition, monocyte chemotactic protein 1 (MCP-1) mRNA expression in HFD mice was significantly higher than the ND mice (*P *<* *0.01). However, this high expression of MCP-1 mRNA was significantly attenuated by exercise training in the HFD mice (*P *<* *0.01, Fig. 1D)

We subsequently investigated the mRNA levels of inflammatory cytokines in the eAT. The mRNA levels of tumor necrosis factor-alpha (TNF-*α*) and interleukin-6 (IL-6) in the eAT in the HFD sedentary mice were higher than those in the ND sedentary mice, and these levels were lower in the HFD exercise mice than in the HFD sedentary mice (Fig. 1D).

### Effects of HFD and exercise training on neutrophil infiltration and elastase expression

**Figure 2:**
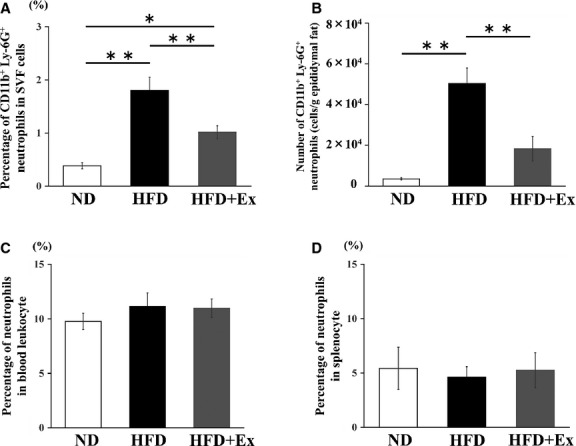
Effect of exercise training on infiltration of neutrophils into the visceral adipose tissue of normal diet- and high-fat-diet-fed mice. (A) Number of CD11b^+^ Ly-6G^+^ cells per gram of epididymal adipose tissue. (B) Percentage of CD11b^+^ Ly-6G^+^ cells in stromal vascular fraction. (C) Percentage of CD11b^+^ Ly-6G^+^ in leukocytes. (D) Percentage of CD11b^+^ Ly-6G^+^ in splenocytes. Values represent mean ± SEM. **P <* *0.01.

We next investigated the total number of neutrophils in the eAT of the mice by flow cytometry for the specific markers CD11b and Ly-6G. Administration of HFD increased the number of neutrophils (4.7-fold) per gram of eAT, and this number was lowered by exercise training (Fig. 2B). When the neutrophil population was analyzed, the percentage of neutrophils comprising the SVF cells of eAT was higher in the HFD sedentary mice than in the ND sedentary or the HFD exercise mice (Fig. 2A). In contrast, the proportion of neutrophils in the isolated leukocyte population and that in the spleen was not different across groups (Fig. 2C and D).

### Effects of HFD and exercise training on NE and IL-8 expression

**Figure 3:**
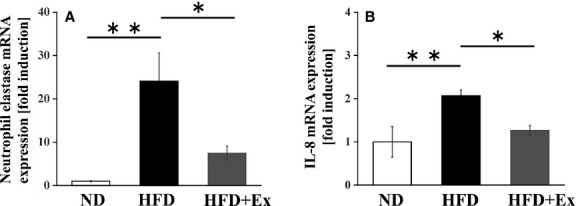
Effect of exercise training on expression of neutrophil elastase and IL-8 in normal diet- and high-fat-diet-fed mice. (A) mRNA expression levels of neutrophil elastase in epididymal adipose tissue (eAT). (B) mRNA expression levels of IL-8 in eAT. Values represent mean ± SEM. *P <* *0.05, **P <* *0.01.

The NE mRNA levels in the eAT were increased by HFD administration in the sedentary mice; however, exercise training attenuated these levels in the HFD mice (Fig. 3A). IL-8 is a major neutrophil chemokine and IL-8, mRNA levels in the eAT were significantly higher in the HFD sedentary mice than in the ND sedentary mice, and these levels were significantly lower in the HFD exercise mice than in the HFD sedentary mice (Fig. 3B).

## Discussion

AT inflammation associated with obesity is one of the key factors for the development of several chronic inflammatory diseases, including type II diabetes and nonalcoholic steatohepatitis (Cornier et al. 2008). AT comprises a variety of cell types including adipocytes, endothelial cells, and immune cells such as macrophages, neutrophils, T cells, and B cells (Chmelar et al. 2013). Immune cells are present in the stromal cell fraction of AT, and obesity changes the proportions of the component immune cells in the SVF (Mathis 2013).

Interestingly, aberrant action of the innate immune response, mainly by macrophages, is important for development of an inflammatory state in local tissues (Lumeng and Saltiel 2011). Therefore, the innate immune response, mainly mediated by macrophages, is key in the inflammatory process of AT. Obese patients display greater numbers of macrophages and neutrophils in AT than normal individuals (Huh et al. 2014). Interestingly, Carmon et al. (2008) reported that neutrophil infiltration into the eAT of mice was observed early in the course of HFD feeding. A recent study showed that neutrophil infiltration was sustained over a longer period after HFD feeding (Talukdar et al. 2012). Neutrophils are known to release macrophage chemoattractants such as MCP-1 and elastase (Soehnlein et al. 2008). NE is responsible for activating and infiltrating macrophages in tissues, which contributes to local tissue inflammation (Soehnlein et al. 2009). Interestingly, a recent study reported that NE-deficient obese mice exhibited reduced infiltration of M1 macrophages and improved AT inflammation (Talukdar et al. 2012). Therefore, NE may play an essential role in the development of AT inflammation by inducing infiltration of M1 macrophages.

Exercise has potent anti-inflammatory effects, and endurance exercise training attenuates local inflammation in AT (Gleeson et al. 2011; Lancaster and Febbraio 2014). Recently, we reported that exercise training in diet-induced obese mice not only decreased macrophage tissue infiltration, but also changed the ratio of macrophage subsets in eAT (Kawanishi et al. 2013). However, the mechanisms by which exercise training induces changes in the number and subset composition of macrophages remain unclear. In this study, we observed that 16 weeks of endurance exercise training reduces the cell number of M1 macrophages in eAT. These results are in agreement with our previous studies (Kawanishi et al. 2013). We analyzed the characteristics of the SVF in eAT by flow cytometry and observed that exercise training decreased the percentage of CD11b^+^ Ly-6G^+^ neutrophils of total SVF cells (HFD sedentary vs. HFD exercise: 1.81 vs. 1.01%), and also reduced the cell number of neutrophils per gram of eAT. Interestingly, this finding was unique to visceral AT because the percentage of neutrophils in the splenocytes and blood leukocytes did not change with HFD feeding and exercise training. These results suggest that the ability of exercise training to suppress AT inflammation and inflammatory macrophage accumulation might be caused by the reduction in neutrophil cell number. We previously reported that exercise training in the absence of a HFD did not affect the macrophage polarization and infiltration in adipose tissue (Kawanishi et al. 2013). However, this study did not include an experimental group for ND plus exercise training. The effects of exercise training in the absence of HFD on neutrophil infiltration in AT are unknown. Further work will be required to determine whether exercise training in the absence of HFD modulates neutrophil infiltration in AT.

Similar to the pattern of neutrophil infiltration, NE mRNA levels were significantly reduced by exercise training. NE can promote infiltration of macrophages into AT via modification of MCP-1 production levels. In fact, an in vitro study demonstrated that NE enhances MCP-1 production from macrophages (Yamaguchi et al. 1999). Moreover, elastase inhibitors inhibit the production of MCP-1 induced by stimulation of endotoxin and inflammatory cytokines (Ishihara et al. 1999). Interestingly, a recent study reported that injection of NE inhibitor into diet-induced obese mice reduced macrophage infiltration in the visceral AT and suppressed AT inflammation (Talukdar et al. 2012). In the present study, exercise training not only decreased NE mRNA levels, but also decreased CD11b^+^ F4/80^+^ macrophage cell numbers and MCP-1 mRNA levels in the eAT. Therefore, the ability of exercise to suppress macrophage infiltration might be caused by the reduction of elastase secreted by neutrophils in obese mice.

Some researchers have reported that exercise training prevents the accumulation of visceral AT following long-term administration of HFD. These results suggest that attenuation in AT inflammation is secondary to changes in AT mass. In this study, 16 weeks of exercise training did not induce the loss of epididymal AT mass in HFD-induced obese mice. Nevertheless, the exercise training markedly inhibited neutrophil infiltration and elastase expression in epididymal AT in obese mice. Our results suggest that exercise training may directly modulate this phenomenon in epididymal AT without requiring exercise training induced loss of epididymal AT mass.

Neutrophils are recruited to various tissues through chemokines and adhesion molecules. Although IL-8 is one of the key chemokines that regulate migration and infiltration of neutrophils, expression of IL-8 is upregulated in the AT of obese patients (Alvehus et al. 2012). Interestingly, Neels et al. (2009) showed that in obese mice deficient in CXCL2, the receptor for IL-8, presented reduced macrophage infiltration in the AT from levels in control obese mice (Neels et al. 2009). In this study, we found that IL-8 mRNA in eAT were significantly lower after exercise training, despite the fact that their expression was increased by the HFD. We also observed that IL-8 mRNA levels in eAT were positively correlated with the number of neutrophils (*r *=* *0.482, *P *<* *0.01) and macrophages (*r *=* *0.556, *P *<* *0.01). Collectively, our findings indicate that the suppression of macrophage and neutrophil infiltration by exercise training may be associated with a reduction in IL-8 levels in obese mice.

In this study, consistent with the decreases in macrophage content of AT, neutrophil content was also markedly lower in mice after 16 weeks of exercise training compared to sedentary mice at the same time point. However, it has not been proven that suppression of neutrophil infiltration by exercise training induced attenuation of macrophage infiltration. Previous studies have shown that neutrophil infiltration precedes accumulation of macrophages in AT obesity (Talukdar et al. 2012). We have examined the effects on the infiltration of neutrophils and macrophages by short-term exercise using 2 week HFD-induced obese mice (unpublished data) and found that macrophage content in eAT was not affected by short-term (2 week) exercise training. Quite surprisingly, neutrophil content in eAT was attenuated by exercise training, suggesting that exercise training attenuates the neutrophil infiltration prior to the macrophage infiltration. It is therefore tempting to speculate that reduction of macrophage infiltration by exercise training is regulated by attenuation of neutrophil infiltration. In human experiments, endurance exercise training gradually reduced the ability of circulating neutrophils to migrate and produce reactive oxygen species and NE in response to consecutive daily exercise sessions (Suzuki et al. 1996, 1999). Future study is necessary to investigate how long it takes to induce these effects of exercise training including the mechanisms of action.

In conclusion, we have demonstrated that exercise training markedly reduces the expression levels of inflammatory cytokines and NE, and attenuates neutrophil and macrophage cell numbers in visceral eAT. Taken together, our results provided evidence that exercise training plays a critical role in reducing macrophage infiltration and AT inflammation by regulating the infiltration of neutrophils.

## Conflict of Interest

None declared.

## Supporting Information

**Table S1.** Primer sequences for real-time RT-PCR analysis. **Figure S1.** Stromal vascular cells from mice were gated according to the side scatter (SSC) and forward scatter plots (FSC) (R1). Neutrophil populations were gated according to CD11b and Ly-6G on the SSC/FSC plot (R2).
