# Supplementary material for: Exercise training attenuates neutrophil infiltration and elastase expression in adipose tissue of high-fat-diet-induced obese mice
Source: Physiol Rep. 2015 Sep 4;3(9):e12534. doi: 10.14814/phy2.12534 (PMC4600380; doi:10.14814/phy2.12534)
Supplement: Supplementary file 1 [file phy20003-e12534-sd1.pptx]

## Slide 1
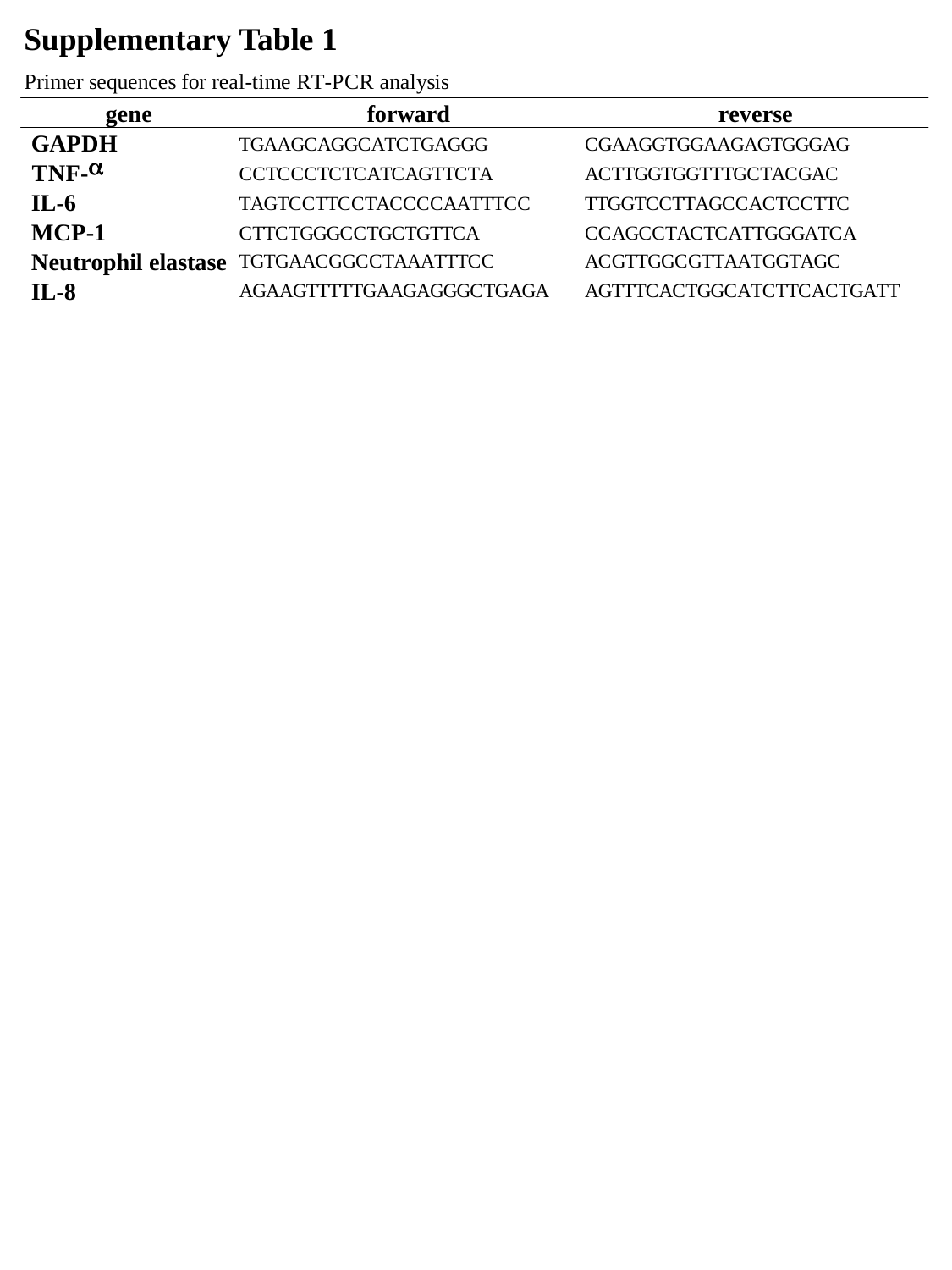

Supplementary Table 1

## Slide 2
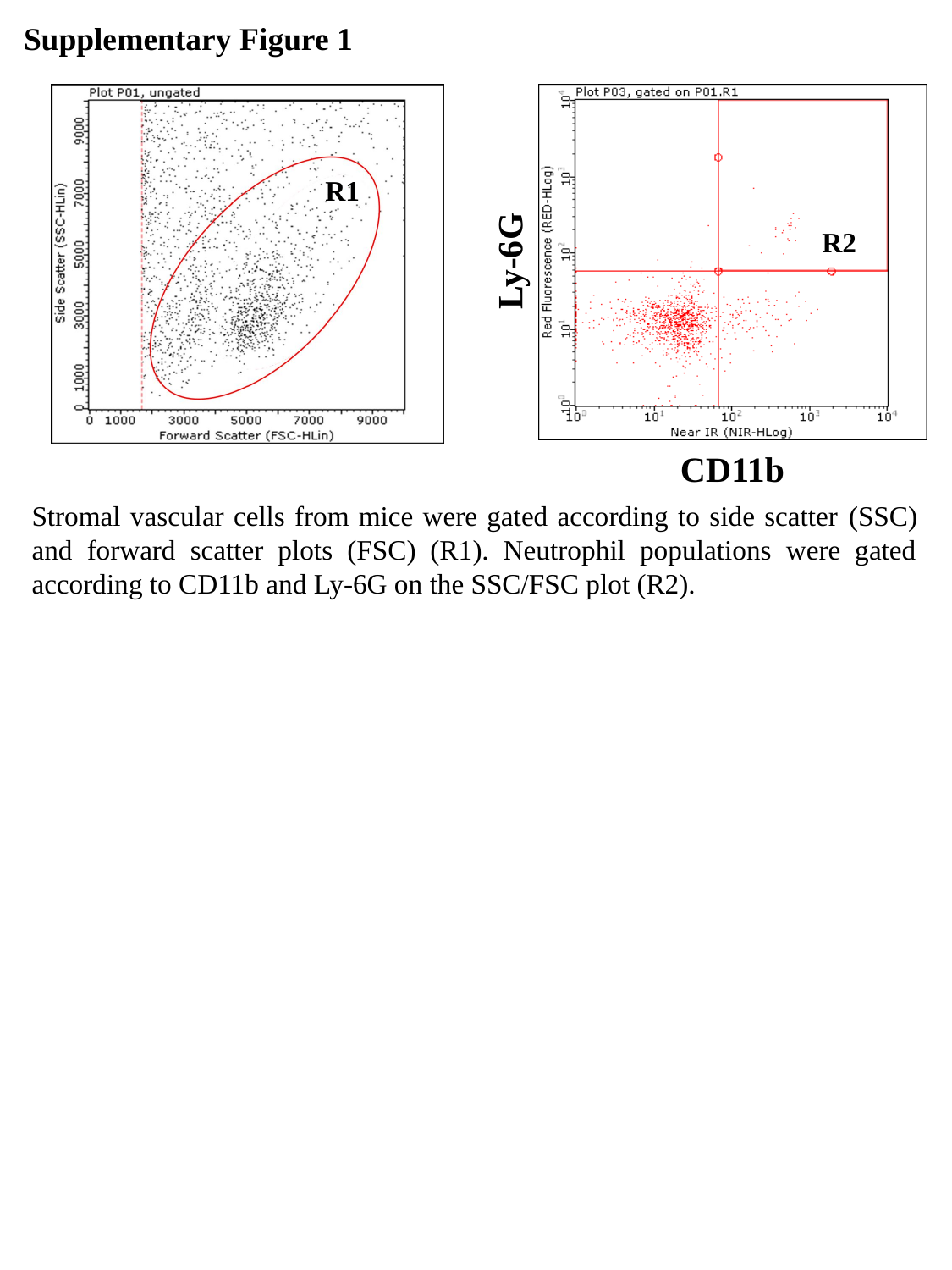

Supplementary Figure 1
R1
R2
Ly-6G
R1
CD11b
Stromal vascular cells from mice were gated according to side scatter (SSC) and forward scatter plots (FSC) (R1). Neutrophil populations were gated according to CD11b and Ly-6G on the SSC/FSC plot (R2).
